# Marine Algae-Derived Bioactive Compounds Stabilizing Collagen-Rich Dental Matrices Through Matrix Metalloproteinase Inhibition: A Scoping Review

**DOI:** 10.3390/md24020071

**Published:** 2026-02-08

**Authors:** Won Sek Lee, Sung-Ae Son, Yong-Il Kim

**Affiliations:** 1 Dental and Life Science Institute, School of Dentistry, Pusan National University, Yangsan 50612, Republic of Korea; wonsekjlee@gmail.com; 2 Department of Conservative Dentistry, School of Dentistry, Pusan National University, Yangsan 50612, Republic of Korea; sung-ae@hanmail.net; 3 Dental Research Institute, Pusan National University Dental Hospital, Yangsan 50612, Republic of Korea

**Keywords:** algae, dentin, matrix metalloproteinase inhibitors, tooth remineralization, collagen type I

## Abstract

This scoping review mapped the available evidence on marine algae-derived bioactive compounds, focusing on their biological activities related to collagen stabilization, matrix metalloproteinase (MMP) inhibition, and enamel remineralization in dental hard tissues. Four electronic databases (PubMed, Scopus, Embase, and Web of Science) were systematically searched following a predefined protocol. Original experimental studies involving human or animal dental hard tissues were included. Nine studies met the inclusion criteria. Brown algal derivatives, including fucoxanthin, fucosterol, and phloroglucinol, exhibited significant MMP inhibition and, in selected compounds, collagen cross-linking, contributing to enhanced mechanical properties and improved stability of collagen-rich matrices. In contrast, red algae extracts such as *Lithothamnion calcareum* primarily promoted enamel remineralization, achieving surface microhardness recovery comparable to or superior to 0.05% sodium fluoride. Alginate, a brown algae-derived polysaccharide, also demonstrated functional potential as a scaffold biomaterial through strong hydroxyapatite adsorption and suitability for three-dimensional scaffold fabrication. Overall, marine algae-derived compounds demonstrate biologically relevant activities that modulate collagen stability, enzymatic function, and mineral deposition processes. These findings highlight the pharmacological potential of marine bioactive compounds, with dental hard tissues representing a primary application context. However, further validation using clinically relevant models is required.

## 1. Introduction

The long-term preservation of mineralized tissues remains a major challenge in various biomedical fields, including oral health. Beyond the simple removal of diseased tissue, maintaining the structural integrity of collagen-rich and mineralized matrices is essential in physiologically hostile environments [1,2]. However, tissue stability is continuously threatened by two interrelated degradative processes: enzymatic collagen breakdown and acid-induced mineral loss [3].

Collagen degradation is primarily mediated by host-derived proteolytic enzymes, particularly matrix metalloproteinases (MMPs) and cysteine cathepsins, which play key roles in extracellular matrix remodeling [4,5,6]. Excessive MMP activity leads to destabilization of collagen fibrils and progressive matrix deterioration. Therefore, identifying bioactive compounds capable of modulating MMP activity and preserving collagen integrity has become an important research focus across multiple biological contexts.

Current therapeutic strategies, although partially effective, do not comprehensively address these concurrent biological degradation pathways. Fluoride—the gold standard for promoting enamel remineralization—primarily reinforces the inorganic phase but offers limited direct protection to the organic collagen matrix [7]. To combat collagen degradation, synthetic inhibitors such as chlorhexidine (CHX) are employed; however, the inhibitory effect of CHX diminishes over time because of leaching, and it notably lacks the ability to mechanically reinforce the dentin structure [8,9]. Furthermore, potent cross-linkers such as glutaraldehyde, although effective at stabilizing collagen, are constrained by cytotoxicity concerns, particularly in deep restorations [10]. Consequently, there is a critical need for bioactive agents capable of simultaneously promoting mineral deposition in dental hard tissues and stabilizing the organic matrix without compromising biocompatibility.

In response to these unmet needs, marine ecosystems—particularly marine algae—have gained attention as a source of bioactive metabolites with demonstrated activity in collagen-based mineralized tissues. Marine ecosystems offer an extensive reservoir of structurally diverse bioactive metabolites with well-documented pharmacological properties [11]. Marine algae-derived compounds—such as fucosterol, fucoxanthin, and alginates—have been widely investigated in orthopedics and tissue engineering owing to their ability to regulate bone metabolism, suppress osteoclastic activity, and modulate inflammatory pathways (e.g., NF-κB and MAPK signaling) [12,13]. These biological activities are particularly relevant to collagen-based mineralized tissues, where the regulation of extracellular matrix degradation and mineral deposition is critical for tissue stability. However, the heterogeneity of algae-derived compounds and their diverse modes of action necessitate clear conceptual definitions to ensure consistent interpretation of their biological roles. This conceptual ambiguity becomes even more pronounced when translating these findings to tissue-specific contexts.

While marine algae-derived compounds have shown promise in other mineralized tissue contexts, their relevance to dental hard tissues remains incompletely understood. In the oral environment, enzymatic degradation of the dentin collagen matrix and acid-induced enamel demineralization contribute to the progressive deterioration of dental hard tissues. Despite the demonstrated efficacy of marine-derived compounds in other mineralized tissue contexts, their potential to modulate collagen stability, MMP activity, and mineral deposition processes in oral tissues remains underexplored [14].

This scoping review aims to address this gap by systematically mapping the current evidence on the application of marine algae-derived bioactive compounds that influence collagen stability, MMP activity, and mineral deposition processes. Specifically, we evaluate their dual potential to remineralize enamel and stabilize the dentin collagen matrix against enzymatic degradation. By synthesizing findings from in vitro and in situ studies, this review seeks to elucidate how the pharmacological properties of marine-derived compounds observed in other biomedical fields may be translated to the preservation of dental hard tissues.

In addition to mapping existing evidence, this review provides a functional systematization of marine algae-derived compounds based on their primary biological roles in dentin matrix stabilization and enamel remineralization. From a translational perspective, these findings highlight the potential of marine-derived bioactives as complementary or alternative strategies to conventional fluoride- and chlorhexidine-based approaches in adhesive dentistry.

To clarify the scope of this review and ensure consistency in interpretation, key terms are defined as follows. In this review, the term marine algae-derived is used to denote compounds that are either directly isolated from marine algal biomass or represent well-established metabolites that naturally occur within marine algae, irrespective of whether they can also be chemically synthesized or identified in non-marine sources. In contrast, compounds that primarily function as structural polymers or carrier materials—such as alginate-based systems—are discussed separately from low-molecular weight bioactive metabolites and are interpreted based on their functional role in delivery or scaffold design rather than intrinsic pharmacological activity.

Within this conceptual framework, it is also necessary to distinguish between the principal biological mechanisms discussed herein. Importantly, MMP inhibition and collagen cross-linking represent mechanistically distinct processes. While both mechanisms may contribute to the preservation of extracellular matrix integrity, MMP inhibition primarily attenuates enzymatic collagen degradation whereas collagen cross-linking enhances the mechanical integrity and degradation resistance of collagen fibrils. In the present review, collagen stabilization is therefore interpreted as a downstream functional outcome that may arise from one or both mechanisms, rather than as an independent or parallel biological process.

## 2. Results

### 2.1. Study Selection and Characteristics

**Figure 1:**
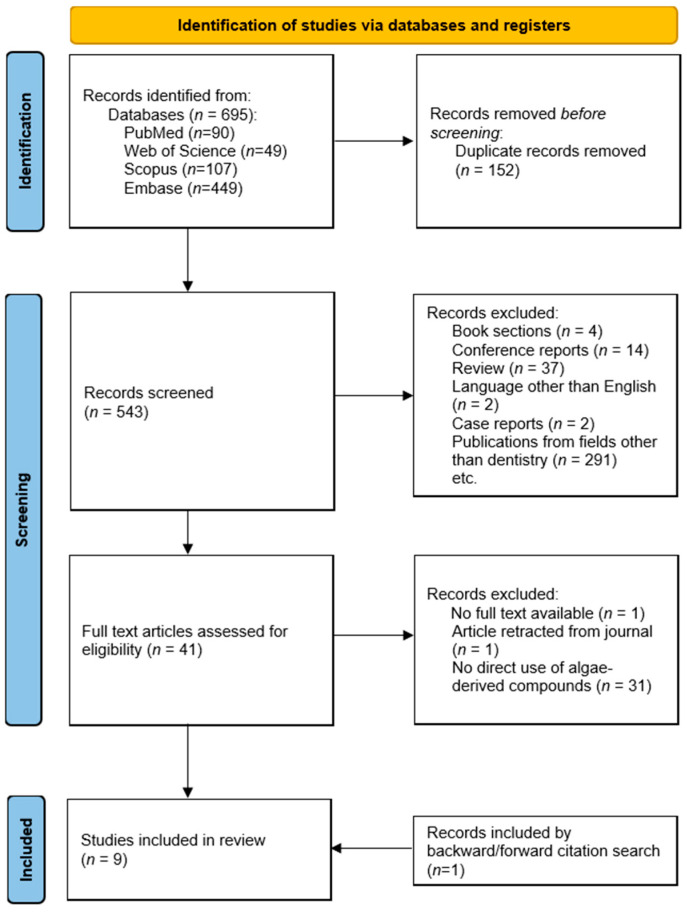
PRISMA flow diagram illustrating the study selection process for this review.

The selection process is illustrated in the PRISMA flow diagram (Figure 1). The search and screening process yielded nine studies that met all eligibility criteria. Table 1 summarizes the characteristics of these studies. All included publications were original experimental research: eight employed exclusively in vitro methodologies, and one utilized a combined in vitro and in situ/ex vivo co-twin control model. No in vivo animal studies were identified. The publication years ranged from 2016 to 2025 (Table 1).

The concepts investigated involved bioactive compounds derived from marine algae, specifically brown algae (fucoxanthin, fucosterol, phloroglucinol, and alginate/propylene glycol alginate) and red algae (*Lithothamnion calcareum* and *Lithothamnion superpositum*) (Table 1).

### 2.2. Synthesis of Results

The findings from the included studies were synthesized into three primary themes: (1) dentin matrix stabilization, encompassing MMP inhibition, collagen cross-linking, and bond durability; (2) enamel mineralization and protection, focusing on remineralization and anti-erosion effects; and (3) marine polymer-based biomaterial systems, including targeted delivery and tissue engineering scaffolds. Table 2 presents a high-level overview of these themes, while Table 3, Table 4 and Table 5 provide detailed comparative data for each theme.

Compounds or systems whose algal origin could not be unequivocally confirmed (e.g., alginate-based scaffolds) were explicitly labeled as marine-occurring or carrier systems and interpreted as supportive mechanistic evidence, consistent with the predefined eligibility framework.

#### 2.2.1. Theme 1: Dentin Matrix Stabilization

Four studies investigated the effects of brown algae-derived compounds (fucoxanthin, fucosterol, and phloroglucinol) on the stabilization of the dentin extracellular matrix (Table 3).

MMP Inhibition: All tested compounds demonstrated significant, concentration-dependent inhibition of MMP activity. In situ zymography visually confirmed this effect for fucoxanthin and fucosterol, whereas fluorometric assays identified phloroglucinol as a particularly potent inhibitor (IC50 = 0.1 mM) [15,16,17,18].

Collagen Stabilization and Mechanical Properties: MMP suppression was associated with enhanced mechanical properties. Fucoxanthin and fucosterol significantly increased the ultimate microtensile strength of demineralized dentin, whereas phloroglucinol significantly increased the apparent flexural elastic modulus [15,16,17,18]. FTIR analyses across these studies confirmed molecular interactions consistent with collagen cross-linking [15,16,17,18].

Adhesive Bond Durability: When applied as primers, these compounds improved the long-term durability of the resin-dentin hybrid layer. Fucoxanthin and fucosterol primers resulted in higher microtensile bond strength and reduced nanoleakage following enzymatic aging with collagenase [15,16,17]. Phloroglucinol primers effectively resisted microtensile bond strength degradation after 12 months of storage [18].

#### 2.2.2. Theme 2: Enamel Mineralization and Anti-Erosion

Three studies investigated the protection of enamel. Table 4 summarizes the comparative findings.

Remineralization: Extracts from the red alga *Lithothamnion calcareum* (LC) demonstrated significant remineralization potential. In a co-twin control in situ study, a 0.03% LC solution achieved a surface microhardness recovery superior to that of 0.05% sodium fluoride [20]. In vitro comparisons also confirmed that LC outperformed *L. superpositum* in promoting hardness recovery [19].

Anti-Erosion: Propylene glycol alginate (PGA), a brown algae-derived compound, was shown to have a synergistic effect with fluoride. The combined fluoride + PGA solution (4.24 µm surface loss [SL]) significantly reduced enamel erosion compared with fluoride alone (5.64 µm SL) [21], as shown in an in vitro erosion-remineralization model (Table 4).

#### 2.2.3. Theme 3: Algae-Derived Polymer-Based Biomaterial Systems

Two studies investigated alginate, a brown algae polysaccharide, not as a bioactive agent but as a structural component for dental applications (Table 5).

Targeting and Scaffolding: Alginate demonstrated utility in targeted delivery, with alginate-coated liposomes showing high affinity for hydroxyapatite in artificial saliva [22]. In tissue engineering, alginate-gelatin-MgHA composite scaffolds successfully mimicked the aligned tubular structure of dentin, supporting the adhesion and proliferation of mesenchymal stem cells [23].

### 2.3. Reporting Limitations

The primary limitation across all studies is the predominance of in vitro designs, which restricts direct clinical translation. Methodological heterogeneity was also noted. Specifically, the use of protein-free artificial saliva and the substitution of intact enamel with hydroxyapatite powder may not fully replicate the complex oral environment. Furthermore, discrepancies were observed in some studies between the concentrations used for bioactivity assays (wt%) and those used for cytotoxicity testing (µg/mL), limiting the precise determination of a therapeutic window.

## 3. Discussion

**Figure 2:**
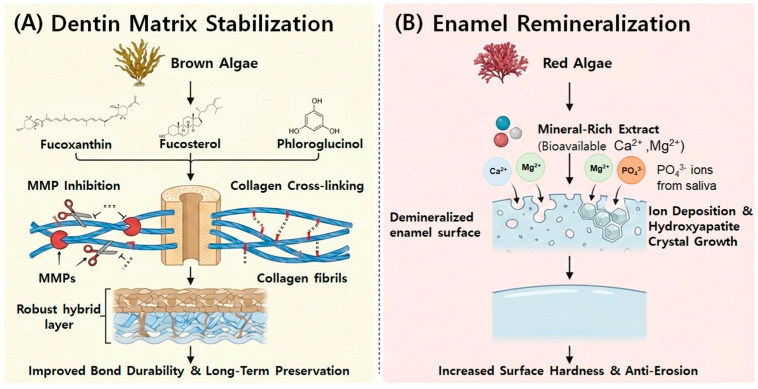
Schematic illustration of the proposed mechanisms of action for marine algae-derived bioactive compounds in dental hard tissue preservation. The diagram delineates the dual pathways: (**A**) the stabilization of the dentin organic matrix via MMP inhibition and collagen cross-linking by brown algae-derived metabolites, and (**B**) the promotion of enamel remineralization through ion deposition and crystal growth by red algae-derived mineral complexes.

This scoping review synthesizes current evidence on marine algae-derived bioactive compounds that modulate collagen stability, enzymatic activity, and mineral deposition in dental hard tissues. As synthesized in Figure 2, the biological activities of these compounds exhibit a distinct functional dichotomy based on the algal source: brown algae-derived metabolites (e.g., fucoxanthin, fucosterol, and phloroglucinol) predominantly function as dentin matrix stabilizers via MMP inhibition and collagen cross-linking, whereas red algae-derived mineral extracts (e.g., *Lithothamnion sp.*) act as potent enamel remineralizing agents. Collectively, these findings suggest that marine-derived compounds may represent a multifunctional biological approach complementary to conventional synthetic agents, potentially overcoming limitations of current gold standards, such as the cytotoxicity of glutaraldehyde or the reversible, non-cross-linking inhibition conferred by CHX [6,14,24]. These bioactivities highlight the potential of marine-derived compounds to modulate extracellular matrix stability and mineral homeostasis, with dental hard tissues representing a primary application context.

To provide a mechanistic synthesis beyond descriptive mapping, the included compounds can be conceptually stratified into three functional categories: (i) low-molecular-weight brown algae-derived metabolites primarily influencing collagen-rich dentin interfaces through attenuation of endogenous proteolytic activity; (ii) red algae-derived mineral complexes acting predominantly on enamel via ion-mediated mineral deposition and crystal growth; and (iii) algae-derived polymers functioning mainly as carrier or scaffold systems with physicochemical rather than intrinsic bioactive effects. Importantly, each category is associated with distinct evidentiary boundaries. Dentin-related studies largely rely on global gelatinolytic assays and bond durability outcomes, limiting inference on specific molecular targets, whereas enamel studies predominantly employ surface-weighted metrics that cannot discriminate superficial mineral precipitation from true subsurface remineralization. Polymer-based systems represent a separate translational class in which observed benefits reflect material–tissue interactions rather than direct biological modulation. This framework clarifies both mechanistic differences and functional limits across algal sources, and highlights where future studies must move beyond surrogate endpoints toward depth-resolved and target-specific analyses.

Throughout this review, the term “collagen stabilization” is used as a collective descriptor encompassing multiple mechanistically distinct phenomena. These include (i) indirect preservation of collagen fibrils via attenuation of endogenous proteolytic activity, most commonly inferred from reduced gelatinolytic signals or improved bond durability; (ii) potential structural reinforcement of the collagen matrix through compound–collagen interactions or altered fibril mechanics; and (iii) apparent stabilization reflected by delayed degradation endpoints under artificial aging conditions. Importantly, most included studies do not directly measure collagen cross-linking or fibrillar architecture, and therefore primarily support functional preservation rather than intrinsic biochemical stabilization. We therefore distinguish between true matrix modification and secondary preservation effects when interpreting reported outcomes.

It is also important to distinguish among the physiological roles of individual matrix metalloproteinase isoforms within dentin. MMP-2 and MMP-9 (gelatinases) are primarily implicated in degradation of denatured collagen and exposed matrix components within the hybrid layer, whereas MMP-8 (collagenase-2) directly cleaves native type I collagen fibrils and is considered a major contributor to dentin collagen breakdown following acid etching [5,6]. However, most studies included in this review infer MMP inhibition using in situ zymography or global gelatinolytic activity assays, which do not resolve isoform-specific contributions. Consequently, the reported outcomes should be interpreted as attenuation of overall proteolytic activity rather than selective inhibition of specific MMP subtypes. This methodological constraint limits mechanistic resolution and highlights the need for future studies employing isoform-targeted assays to clarify compound-specific effects on dentin matrix degradation.

A key finding of this review is that fucosterol and fucoxanthin substantially attenuate overall gelatinolytic activity in human dentin models [15,16,17]. Although their precise intracellular mechanisms of action within the dentin microenvironment remain unclear, the available data support a functionally meaningful suppression of endogenous proteolysis, consistent with reduced gelatinolytic activity and improved bond durability reported in the included studies. In the context of dental hard tissues, the primary biologically relevant outcome of these compounds is preservation of the collagen-rich matrix and stabilization of the hybrid layer. Mechanistic insights from broader biomedical literature, including NF-κB/MAPK-linked regulation of MMP expression, are therefore best interpreted as supportive biological context rather than direct evidence of oral tissue-specific regulatory pathways [13,25,26,27,28].

Although several studies compare algae-derived compounds with CHX using surrogate outcomes such as gelatinolytic activity or aged bond strength, these agents operate within fundamentally different mechanistic and clinical paradigms. CHX functions primarily as a broad-spectrum antimicrobial and direct protease inhibitor, providing short-term suppression of enzymatic degradation at the dentin–resin interface [8,9,29]. In contrast, algae-derived metabolites appear to exert indirect matrix-preserving effects, potentially mediated through antioxidant activity, modulation of cellular signaling pathways, or alterations in collagen susceptibility to degradation. Accordingly, similar experimental outcomes should not be interpreted as mechanistic equivalence. Rather, algae-derived compounds may be better viewed as exploratory adjunctive agents with matrix-modulatory potential, distinct from chlorhexidine’s established antimicrobial and enzymatic inhibition profile. Importantly, chlorhexidine has been widely investigated and applied as an adjunctive agent in adhesive dentistry to transiently suppress dentin matrix degradation, whereas algae-derived compounds remain at a preclinical stage, requiring further validation of dosing, delivery strategies, and clinical feasibility [6,8,29].

Despite these fundamental mechanistic differences, collagen-rich interfacial matrices remain vulnerable to enzymatic degradation, particularly collagen hydrolysis. Unlike CHX, which functions as a passive and reversible inhibitor that leaches over time [8,29], marine-derived compounds were associated with sustained matrix stability following enzymatic aging in the included studies [15,17]. However, direct comparative evidence between CHX and algae-derived compounds remains limited, and the observed differences should be interpreted in the context of distinct experimental designs and outcome measures. Furthermore, the cross-linking capability of phloroglucinol, supported by FTIR spectral shifts consistent with new bond formation observed in this review [18], likely arises from hydrophobic interactions and hydrogen bonding with collagen fibrils [14,30]. This biologically mediated matrix stabilization provides mechanical reinforcement via a mechanism distinct from synthetic aldehydes [31], enabling complementary protective mechanisms involving enzymatic inhibition and, in selected compounds, structural strengthening.

With respect to enamel protection, the red alga *Lithothamnion calcareum* demonstrated enamel remineralization efficacy comparable to or superior to that of fluoride [19,20]. This superiority is attributed to its unique, highly porous lattice structure and the synergistic presence of trace elements, particularly magnesium [32]. Magnesium is a critical modulator of hydroxyapatite crystal growth, stabilizing the amorphous calcium phosphate precursor and preventing the formation of brittle crystals [33,34,35]. However, remineralization outcomes in these studies were primarily evaluated using surface-based metrics, such as surface microhardness and mineral content, which do not capture subsurface lesion repair or interfacial mineralization patterns—critical determinants of long-term enamel stability. Accordingly, it remains unclear whether the observed mineral gains reflect superficial precipitation or deeper structural remineralization. This represents a key methodological limitation, as surface-based assessments cannot reliably discriminate superficial mineral deposition from true subsurface lesion repair. From a biomineralization perspective, the marine mineral complex may theoretically support subsurface mineral deposition by mimicking the stoichiometric complexity of biological apatite, rather than merely forming a superficial fluorapatite layer, as seen with topical fluorides [7]. These characteristics underscore the biological relevance of marine mineral extracts in regulating mineral deposition processes.

Beyond low-molecular-weight metabolites, marine polysaccharides also contribute to dental applications through their role as structural biomaterials. The use of alginate to fabricate scaffolds with aligned microporosity [23] illustrates the versatility of these biopolymers in replicating hierarchical tissue architecture. Anisotropy is essential in tissue engineering for guiding cellular orientation and regulating nutrient transport [36,37,38]. The recapitulation of dentinal tubule architecture is particularly crucial for functional dentin-pulp complex regeneration. Alginate-based systems, with their tunable cross-linking and hydrophilicity, offer a distinct advantage over synthetic polymers by supporting mesenchymal stem cell adhesion while maintaining structural anisotropy [39,40,41]. Importantly, these outcomes reflect the physicochemical and architectural properties of alginate rather than intrinsic biological activity. Accordingly, alginate is interpreted herein as a carrier or scaffold biomaterial platform, distinct from low-molecular weight marine algae-derived metabolites with demonstrated bioactive effects.

From a mechanistic perspective, none of the included studies systematically evaluated selective inhibition of individual MMP subtypes (e.g., MMP-2, MMP-8, or MMP-9) using subtype-resolved enzymatic assays. Although in situ zymography employing quenched fluorescein-conjugated gelatin suggests suppression of gelatinase activity—primarily associated with MMP-2 and MMP-9—and in silico docking analyses have proposed potential interactions with these enzymes [15,16,17], the reported outcomes were based predominantly on global gelatinolytic activity rather than direct functional inhibition of specific MMP isoforms. While these findings support overall attenuation of endogenous proteolysis, they do not permit definitive conclusions regarding subtype-selective biological effects. Future studies incorporating recombinant enzyme assays or subtype-specific activity profiling will be essential to clarify biological specificity and translational relevance. Importantly, the observed global suppression of gelatinolytic activity remains biologically meaningful in collagen-rich interfacial environments, where cumulative proteolysis plays a central role in matrix degradation.

Despite these promising insights, translation into clinical dentistry remains challenging. Most included studies rely on in vitro models (Table 1), which lack the physiological complexity of pulpal pressure, saliva flow, and multispecies biofilms. Furthermore, improvements in bond durability were primarily inferred from aged MTBS, which represents an indirect surrogate of resin–dentin interfacial integrity and is highly sensitive to methodological heterogeneity. Variables such as tooth substrate, including the use of human versus bovine teeth, specimen dimensions, and testing parameters (e.g., crosshead speed), can introduce confounding effects on reported bond strengths. Aging protocols also varied substantially, ranging from simple water storage to enzymatic challenges or thermocycling, none of which fully replicate the dynamic and multifactorial degradation processes occurring intraorally. Accordingly, durability observed under specific laboratory configurations may not consistently reflect clinical performance, underscoring the need for standardized testing protocols in future translational research. It should therefore be emphasized that MTBS should not be regarded as a direct predictor of long-term clinical performance. A notable example of these constraints is the synergistic anti-erosive effect of PGA combined with fluoride, which has been demonstrated only under simplified in vitro conditions. These models often fail to replicate critical clinical variables, such as the protective role of the acquired pellicle—a proteinaceous film that acts as a diffusion barrier—as well as salivary clearance and the oral cavity’s complex buffering capacity.

Another critical translational limitation identified in this review is the narrow therapeutic window. Specifically, concentrations required to achieve meaningful attenuation of dentin gelatinolytic activity closely overlap with levels associated with cytotoxic responses in dental pulp-derived cells, creating a narrow margin for safe biological modulation. As demonstrated in this review [15,17], fucoxanthin and fucosterol concentrations required for maximal MMP inhibition in dentin bonding approached or exceeded cytotoxic levels for human dental pulp stem cells. This discrepancy highlights the need for precise dosage calibration. Future delivery systems, potentially utilizing the liposomal encapsulation strategies identified in this review [22], must ensure localized bioactivity while minimizing off-target cytotoxicity [42,43]. No experimental studies involving green algae were identified in our systematic search that directly evaluated dental hard tissues. This underrepresentation may reflect historical research focus on brown and red algae, which have historically received greater attention in biomedical research owing to their more extensively characterized polyphenolic metabolites and mineral-associated components. Nevertheless, green algae possess distinct metabolite profiles, including sulfated polysaccharides and bioactive pigments, suggesting untapped potential for dental biomaterial development. Systematic investigation of green algae-derived compounds may therefore represent a promising direction for future translational research. This absence reflects a current gap in the literature and highlights an opportunity for future investigations into green algae-derived compounds in dental applications. In parallel, future studies should assess compatibility with contemporary adhesive systems and clinically realistic application protocols such as primer incorporation, application time, and post-treatment rinsing, alongside standardized biocompatibility testing to facilitate translational development.

## 4. Materials and Methods

A scoping review summarizes existing data based on a predefined protocol to provide a descriptive overview of the literature [44,45].

### 4.1. Protocol and Registration

The protocol for this scoping review was developed a priori and registered on the Open Science Framework (OSF; https://osf.io/95qzm, accessed on 7 February 2026). This review was conducted in accordance with the Joanna Briggs Institute (JBI) methodology for scoping reviews and reported following the Preferred Reporting Items for Systematic Reviews and Meta-Analyses Extension for Scoping Reviews (PRISMA-ScR) guidelines.

### 4.2. Search Strategy

The research team, including a medical librarian, first established the research questions and relevant keywords. A peer-reviewed search strategy [46,47] was conducted in November 2025 across four databases (PubMed, Scopus, Embase, and Web of Science) using terms related to “dentistry” and “marine algae” (Full search strategies are provided in Tables S1–S4).

### 4.3. Study Selection and Screening

Search results were imported into EndNote (version 21.5; Clarivate Analytics, Philadelphia, PA, USA) for duplicate removal. Using the evidence Review Accelerator (TERA) [48], two reviewers independently screened all titles and abstracts based on predefined inclusion and exclusion criteria (Tables S5–S8). Two additional reviewers then independently assessed the full texts of all potentially eligible articles. A third reviewer resolved any conflicts to determine the final inclusion.

### 4.4. Data Extraction and Scope

One reviewer extracted data from the final included studies into structured tables (Table 1, Table 2, Table 3, Table 4 and Table 5). This review was limited to original experimental research; gray literature was excluded. Reference list screening and contact with authors or subject matter experts were not undertaken.

## 5. Conclusions

This scoping review synthesizes current evidence on marine algae-derived compounds as modulators of collagen stability, enzymatic activity, and mineral deposition in dental hard tissues. The available data indicate that brown algae-derived metabolites may contribute to stabilization of collagen-rich matrices primarily through inhibition of endogenous matrix metalloproteinase activity and, in some cases, collagen cross-linking, whereas red algae-derived mineral extracts show promise for enhancing enamel remineralization. Together, these findings highlight the biological and pharmacological relevance of marine-derived compounds in regulating extracellular matrix integrity and biomineralization processes, with oral tissues representing one of several potential application fields.

Despite encouraging experimental outcomes, several critical translational bottlenecks remain. Improvements in bond durability were largely inferred from indirect surrogate endpoints such as aged MTBS, which is highly sensitive to experimental variables and therefore does not necessarily predict long-term clinical performance; mechanistic specificity at the level of individual MMP subtypes also remains insufficiently characterized. In addition, the therapeutic window of key compounds such as fucoxanthin and fucosterol appears narrow, with concentrations required for maximal MMP inhibition approaching cytotoxic thresholds in human dental pulp stem cells. With respect to enamel protection, the reported synergistic anti-erosive effects of PGA combined with fluoride are currently supported only by in vitro evidence, and no experimental studies involving green algae were identified that directly evaluated dental hard tissues, reflecting a current gap in the literature. Notably, all included studies were conducted in vitro, with only one incorporating in situ/ex vivo components, underscoring the current absence of in vivo evidence.

A critical translational limitation is that dentin application concentrations reported in several studies approach or exceed levels associated with cytotoxic responses in dental pulp-derived cells, as evidenced by available human dental pulp stem cell data for fucosterol. This narrow margin between biologically effective and cytotoxic concentrations underscores the importance of defining compound-specific therapeutic windows and balancing biological efficacy against cellular safety prior to clinical translation.

Furthermore, most available data derive from simplified in vitro models that do not account for critical oral factors such as acquired pellicle formation, salivary clearance, multispecies biofilms, and dynamic pH buffering. Addressing these limitations will require standardized testing protocols, depth-resolved mineralization analyses, subtype-specific enzymatic assays, and in vivo validation, together with clinically feasible delivery strategies. Overall, overcoming these challenges will be essential for translating marine algae-derived compounds into predictable and biologically informed dental therapies.

## Figures and Tables

**Table 1:** Overview of included studies.

Study (Author, Year)	Algae Type	Bioactive Compound/Material	Experimental Model
Lee et al. (2025) [15]	Brown	Fucoxanthin	In vitro (Human dentin)
Lee et al. (2024) [16]	Brown	Fucosterol, Fucoxanthin	In vitro (Human dentin)
Kim et al. (2024) [17]	Brown	Fucosterol	In vitro (Human dentin)
Yoo et al. (2024) [18]	Brown	Phloroglucinol, Marine Chitosan	In vitro (Human dentin)
Chakravarthy et al. (2023) [19]	Red	*Lithothamnion calcareum*, *L. superpositum*	In vitro (Human enamel)
Carrilho & Bretz (2023) [20]	Red	*Lithothamnion calcareum*	In vitro & In situ/Ex vivo (Bovine/Human enamel)
Bezerra et al. (2019) [21]	Brown	Propylene glycol alginate (PGA)	In vitro (Bovine enamel/dentin)
Pistone et al. (2016) [22]	Brown	Alginate	In vitro (Liposome/HA powder)
Panseri et al. (2016) [23]	Brown	Alginate	In vitro (3D Scaffold/MSCs)

**Table 2:** Thematic Overview of Algae-Derived Compounds and Primary Effects.

Theme [Ref]	Mechanism	Representative Compounds/Systems (Algae Source)	Key Assessment Metrics	Key Results (Effect)
**1. Dentin Matrix Stabilization [15,16,17,18]**	**MMP Inhibition** (Enzymatic inhibition)	**Fucoxanthin** (Brown algae)	In situ zymography	**↑** MMP Inhibition (Dose-dependent)
		**Fucosterol** (Brown algae)		
		**Phloroglucinol** (Brown algae)	Fluorometric assay	
	**Collagen Stabilization** (Cross-linking & mechanics)	**Fucoxanthin** (Brown algae)	FTIR (Cross-linking)	**↑** Mechanical properties (µUTS, AE)
		**Fucosterol** (Brown algae)	µUTS (Tensile strength)	(FTIR confirmed cross-linking)
		**Phloroglucinol** (Brown algae)	AE (Flexural modulus)	
	**Bond Durability** (Hybrid layer)	**Fucoxanthin** (Brown algae)	MTBS (Aged)	**↑** Long-term bond strength retention
		**Fucosterol** (Brown algae)	Nanoleakage (SEM)	**↓** Nanoleakage
		**Phloroglucinol** (Brown algae)	-	-
**2. Enamel Mineralization & Protection [19,20]**	**Remineralization** (Mineral gain)	***Lithothamnion calcareum*** (*Red algae*)	Microhardness (Vickers, Knoop)	**↑** Microhardness recovery (≥NaF)
		***L. superpositum*** (*Red algae*)	QLF (Mineral gain)	**↑** Mineral gain
			XRF (Ca/P content)	
	**Anti-Erosion**	**Propylene glycol alginate (PGA)** (Brown algae)	Optical Profilometry (SL)	**↓** Surface Loss (Synergistic with Fluoride)
**3. Biomaterial Systems [21,22,23]**	**Dental Targeting**	**Alginate** (Brown algae)	HA Adsorption Assay (Fluorescence)	**↑** HA (enamel model) adsorption
	**Tissue Engineering**	**Alginate** (Brown algae)	SEM, 3D Cell Culture (MSCs)	**↑** Cell adhesion & proliferation

↑ indicates an increase compared with control; ↓ indicates a decrease compared with control.

**Table 3:** Comparative Summary of Dentin Matrix Stabilization (MMP Inhibition, Mechanics, Bond Durability).

Compound (Algae Source) [Ref]	Model	MMP Inhibition Results	Mechanical Property Results	Bond Durability Results (Aged)
Fucoxanthin (Brown algae) [15,16]	In vitro (Human dentin)	Dose-dependent inhibition (Zymography)	Significantly increased µUTS	Improved MTBS/ Reduced Nanoleakage
Fucosterol (Brown algae) [16,17]	In vitro (Human dentin)	Dose-dependent inhibition (Zymography)	Significantly increased µUTS	Improved MTBS/ Reduced Nanoleakage
Phloroglucinol (Brown algae) [18]	In vitro (Human dentin)	Strong inhibition (IC_50_ = 0.1 mM)	Significantly increased AE	Resisted MTBS degradation (12-mo)

µUTS: Ultimate Microtensile Strength. AE: Apparent Flexural Elastic Modulus. MTBS: Microtensile Bond Strength.

**Table 4:** Comparative Summary of Enamel Remineralization and Anti-Erosive Effects.

Compound (Algae Source) [Ref]	Model	Assessment Methods	Key Results (vs. Control)
*Lithothamnion calcareum* (Red algae) [19]	In vitro (Artificial enamel lesion)	Vickers Microhardness; XRF	*L. calcareum* > *L. superpositum*. (Both lower than CPP-ACPF control)
*Lithothamnion superpositum* (Red algae) [19]			
*Lithothamnion calcareum* (Red algae) [20]	In vitro (pH-cycling)	Knoop Microhardness (%SHR); QLF; Mass Change	Equivalent or Superior to 0.05% NaF. (%SHR significantly higher than NaF in situ)
	In situ/Ex vivo (Co-twin)		
Propylene glycol alginate (PGA) (Brown algae) [21]	In vitro (Erosion-remineralization)	Optical Profilometry (SL)	Synergistic effect with Fluoride (F). (F + PGA significantly reduced Surface Loss vs. F alone)

%SHR: Surface Microhardness Recovery. QLF: Quantitative Light-induced Fluorescence. XRF: X-ray Fluorescence Spectroscopy. SL: Surface Loss.

**Table 5:** Algae-Derived Polymer-Based Biomaterial Systems.

Compound (Algae Source)	System Type [Ref]	Model	Key Results (System Properties)
Alginate (Brown algae)	Liposomal coating for drug delivery [22]	In vitro (HA powder adsorption)	High adsorption to HA (enamel model) in artificial saliva; stable formulation
	3D Biomimetic Scaffold [23]	In vitro (MSCs 3D culture)	Created aligned porous structure mimicking dentinal tubules; enhanced MSC adhesion and proliferation

HA: Hydroxyapatite. MSC: Mesenchymal stem cell.

## Data Availability

The data that support the findings of this study are available from the corresponding author upon reasonable request.

## Supplementary Materials

The following supporting information can be downloaded at: https://www.mdpi.com/article/10.3390/md24020071/s1, Table S1. PubMed Search; Table S2. Embase Search; Table S3. Scopus Search; Table S4. Web of Science Search; Table S5. Population inclusion and exclusion criteria with examples; Table S6. Concept inclusion and exclusion criteria with examples.; Table S7. Context inclusion and exclusion criteria with examples.; Table S8. Main themes to be studied with description.

## Abbreviations

The following abbreviations are used in this manuscript:

MMP	Matrix Metalloproteinase
CHX	Chlorhexidine
µUTS	Ultimate Microtensile Strength
AE	Apparent Flexural Elastic Modulus
MTBS	Microtensile Bond Strength
%SHR	Surface Microhardness Recovery
QLF	Quantitative Light-induced Fluorescence
XRF	X-ray Fluorescence Spectroscopy
SL	Surface Loss
HA	Hydroxyapatite
MSC	Mesenchymal stem cell
LC	*Lithothamnion calcareum*
NaF	Sodium Fluoride
F	Fluoride
PGA	Propylene glycol alginate
CPP	Casein phosphopeptide
ACPF	Amorphous calcium phosphate fluoride
