# Supplementary material for: Marine Algae-Derived Bioactive Compounds Stabilizing Collagen-Rich Dental Matrices Through Matrix Metalloproteinase Inhibition: A Scoping Review
Source: Mar Drugs. 2026 Feb 8;24(2):71. doi: 10.3390/md24020071 (PMC12942173; doi:10.3390/md24020071)
Supplement: Supplementary file 1 [file marinedrugs-24-00071-s001.zip › marinedrugs-4119685-supplementary.pdf]

Supplementary Material for: Marine Algae-Derived Bioactive Compounds Stabilizing Collagen-Rich Dental Matrices Through Matrix Metalloproteinase Inhibition: A Scoping Review

Table S1. PubMed Search<br>*Database: PubMed All to November 1, 2025*<br>*Date searched: November 1, 2025*				
	#	Search Details	Results	P/C/Cx
→ **PubMed**	1	"dentin"[MeSH Terms] OR "dentin"[Title/Abstract] OR "dentin"[Text Word] OR "Dentine"[Text Word] OR "Dentines"[Text Word] OR "dentinal"[Text Word] OR "dentinum"[Text Word] OR "tooth"[Title/Abstract] OR "teeth"[Title/Abstract] OR "dental"[Title/Abstract]	468,942	**P**
	2	"dental enamel"[MeSH Terms] OR "Enamel"[Text Word] OR "Enamels"[Text Word] OR "dental hard tissue"[Text Word]	43,524	
	3	"tooth demineralization"[MeSH Terms] OR "demineralization tooth"[Text Word] OR "decalcification*"[Text Word] OR "demineralі*"[Text Word] OR "dental demineralization"[Text Word]	71,057	
	4	"tooth erosion"[MeSH Terms] OR "erosion tooth"[Text Word] OR "tooth erosion*"[Text Word] OR "dental erosion"[Text Word] OR "dental erosions"[Text Word] OR "erosion dental"[Text Word] OR "dental enamel erosion"[Text Word] OR "dental enamel erosions"[Text Word] OR "enamel erosion"[Text Word] OR "erosion dental enamel"[Text Word] OR "eroded teeth"[Text Word] OR "eroded tooth"[Text Word]	4,000	
	5	"collagen type i"[MeSH Terms] OR "type 1 collagen"[Text Word] OR "collagen type 1"[Text Word] OR "type i collagen"[Text Word] OR "collagen type i"[Text Word] OR "collagen 1"[Text Word] OR "collgen i"[Text Word] OR "collagen type 1"[Text Word]	42,052	
	6	"matrix metalloproteinase 9"[MeSH Terms] OR "metalloproteinase 9 matrix"[Text Word] OR "92 kda type iv collagenase"[Text Word] OR "92 kda type iv collagenase"[Text Word] OR "matrix metalloproteinase 9"[Title/Abstract] OR "92 kda gelatinase"[Text Word] OR "mmp 9 metalloproteinase"[Text Word] OR "metalloproteinase mmp 9"[Text Word] OR "mmp 9 metalloproteinase"[Text Word] OR "mmp9 metalloproteinase"[Text Word] OR "metalloproteinase mmp9"[Text Word] OR "gelatinase b"[Text Word] OR "MMP9"[Text Word] OR "MMP-9"[Title/Abstract] OR "MMP-9"[Text Word]	45,001	
	7	"matrix metalloproteinase 2"[MeSH Terms] OR "metalloproteinase 2 matrix"[Text Word] OR "72 kda type iv collagenase"[Text Word] OR "72 kda type iv collagenase"[Text Word] OR "matrix metalloproteinase 2"[Title/Abstract] OR "72 kda gelatinase"[Text Word] OR "gelatinase 72 kda"[Text Word] OR "mmp 2 metalloproteinase"[Text Word] OR "metalloproteinase mmp 2"[Text Word] OR "mmp 2 metalloproteinase"[Text	34,160	

		Word] OR "mmp2 metalloproteinase"[Text Word] OR "metalloproteinase mmp2"[Text Word] OR "gelatinase a"[Text Word] OR "MMP-2"[Title/Abstract] OR "MMP2"[Title/Abstract] OR "type iv collagenase"[Text Word]		
	8	"collagenases"[MeSH Terms] OR "collagenase*"[Title/Abstract] OR "collagen peptidase"[Text Word] OR "collagen degrading enzyme"[Text Word] OR "nucleolysin"[Text Word]	62,629	
	9	"gelatinases"[MeSH Terms] OR "Gelatinase"[Text Word] OR "gelatinases"[Text Word]	41,001	
	10	#1 OR #2 OR #3 OR #4 OR #5 OR #6 OR #7 OR #8 OR #9	**633,146**	
	11	"phlorotannin*"[Text Word] OR "phlorotannin*"[Title/Abstract]	659	**C**
	12	"fucophlorethol*"[Text Word] OR "fucophlorethol*"[Title/Abstract]	17	
	13	"eckol*"[Text Word] OR "eckol*"[Title/Abstract]	106	
	14	"fucan sulfate*"[Text Word] OR "fucoidin*"[Text Word] OR "sulfated fucan*"[Text Word] OR "fucan sulfate hor 1"[Text Word] OR "mekabu fucoidan"[Text Word] OR "meFucoidan"[Text Word] OR "fucoidan*"[Text Word] OR "fucan sulfate*"[Title/Abstract] OR "fucoidin*"[Title/Abstract] OR "fucoidan*"[Title/Abstract]	3,612	
	15	"laminarin*"[Text Word] OR "laminaran*"[Text Word] OR "laminarin*"[Title/Abstract] OR "laminaran*"[Title/Abstract]	1,565	
	16	"phytosterols"[MeSH Terms] OR "plant sterol*"[Text Word] OR "sterol plant"[Text Word] OR "plant steroid*"[Text Word] OR "steroid plant"[Text Word] OR "phytosteroid*"[Text Word] OR "sterols plant"[Text Word] OR "phytosterol*"[Text Word] OR "steroids plant"[Text Word]	14,005	
	17	"fucoxanthin*"[Text Word] OR "fucoxanthin*"[Title/Abstract]	1,416	
	18	"avenasterol*"[Text Word] OR "28-isofucosterol"[Text Word] OR "fucosterol*"[Text Word] OR "avenasterol*"[Title/Abstract] OR "fucosterol*"[Title/Abstract]	340	
	19	"phaeophyceae"[MeSH Terms] OR "phaeophyta*"[Text Word] OR "algae brown"[Text Word] OR "brown alga*"[Text Word] OR "fucophyceae"[Text Word] OR "phaeophyceae*"[Text Word] OR "phaeophyt*"[Text Word] OR "seaweed*"[Text Word]	17,006	
	20	"alga*"[Text Word]	70,169	
	21	"undaria"[MeSH Terms] OR "wakame*"[Text Word] OR "alginate*"[Text Word]	33,822	
	22	"kelp"[MeSH Terms] OR "saccharina japonica"[Text Word] OR "laminaria japonica"[Text Word] OR "konbu	4,618	

		kelp*"[Text Word] OR "dasima*"[Text Word] OR "haidai*"[Text Word] OR "kombu*"[Text Word] OR "Kjellmaniella"[Text Word] OR "Saccharina"[Text Word] OR "miyeok*"[Text Word] OR "kelp*"[Text Word] OR "konbu*"[Text Word]		
	23	"seaweed"[MeSH Terms] OR "Macroalgae"[Text Word]	9,206	
	24	"chlorophyta"[MeSH Terms] OR "green algae"[Text Word] OR "algae green"[Text Word] OR "Chlorophytina"[Text Word]	30,192	
	25	"rhodophyta"[MeSH Terms] OR "algae red"[Text Word] OR "red algae"[Text Word]	6,066	
	26	"Carrageenan"[MeSH Terms] OR "Carrageenin"[Text Word] OR "iota-Carrageenan"[Text Word] OR "iota-Carrageenan"[Text Word] OR "kappa-Carrageenan"[Text Word] OR "kappa-Carrageenan"[Text Word] OR "lambda-Carrageenan"[Text Word] OR "lambda-Carrageenan"[Text Word]	10,981	
	27	#11 OR #12 OR #13 OR #14 OR #15 OR #16 OR #17 OR #18 OR #19 OR #20 OR #21 OR #22 OR #23 OR #24 OR #25 OR #26	**154,100**	
	28	"dentin bonding*"[Text Word]	8,019	**Cx**
	29	"dentin bonding agents"[MeSH Terms] OR "dentin bonding agent*"[Text Word]	7,741	
	30	"bond strength*"[Text Word]	17,480	
	31	"matrix metalloproteinase inhibitors"[MeSH Terms] OR "mmp inhibitor*"[Text Word] OR "collagenase inhibitor*"[Text Word] OR "gelatinase inhibitor*"[Text Word] OR "stromelysin inhibitor*"[Text Word] OR "matrix metalloproteinase inhibitor*"[Text Word] OR "mmp inhibit*"[Text Word]	8,405	
	32	"Tooth Remineralization"[MeSH Terms] OR "tooth remineralization*"[Text Word] OR "remineraliz*"[Text Word] OR "remineralis*"[Text Word]	6,248	
	33	"collagen crosslink*"[Text Word] OR "crosslink*"[Text Word]	141,980	
	34	#28 OR #29 OR #30 OR #31 OR #32 OR #33	**177,416**	
	35	#10 AND #27 AND #34	**148**	
	36	"bone and bones"[MeSH Terms] OR ("Bone"[Text Word] AND "Bone"[Text Word]) OR ("Bones"[Text Word] AND "Bone"[Text Word]) OR ("Bones"[Text Word] AND "bone tissue"[Text Word]) OR "Bones"[Text Word] OR "Bone"[Text Word] OR "bone tissue"[Text Word] OR "bone tissues"[Text Word] OR "tissue bone"[Text Word] OR "tissues bone"[Text Word] OR "bony apophyses"[Text Word] OR (("apophyseal"[All Fields] OR "Apophyses"[All Fields] OR "apophysitis"[All Fields]) AND "Bony"[Text Word]) OR ("Bony"[All Fields] AND "Apophysis"[Text Word]) OR ("Apophysis"[All Fields] AND "Bony"[Text	1,560,866	**Lim**

		Word]) OR "Condyle"[Text Word] OR "Condyles"[Text Word]		
	37	"Hydrogels"[MeSH Terms] OR "Hydrogel"[Text Word] OR "in situ hydrogels"[Text Word] OR "in situ hydrogel"[Text Word] OR "hydrogel in situ"[Text Word] OR "patterned hydrogels"[Text Word] OR "patterned hydrogel"[Text Word] OR "hydrogel patterned"[Text Word]	75,546	
	38	"cartilage"[MeSH Terms] OR "Cartilages"[Text Word]	103,558	
	39	#35 NOT #36 NOT #37 NOT #38	**74**	

Table S2. Embase Search
Database: Embase All to November 1, 2025
Date searched: November 1, 2025

	#	Query	Results	P/C/Cx
→ **Embase**	#01	dentin'/exp OR 'dentin':ti,ab,kw OR 'dentin':ti,ab,kw,de,dn,df,mn,tn OR 'dentine':ti,ab,kw,de,dn,df,mn,tn OR 'dentines':ti,ab,kw,de,dn,df,mn,tn OR 'dentinal':ti,ab,kw,de,dn,df,mn,tn OR 'dentinum':ti,ab,kw,de,dn,df,mn,tn OR 'tooth':ti,ab,kw OR 'teeth':ti,ab,kw OR 'dental':ti,ab,kw	495341	**P**
	#02	enamel'/exp OR 'enamel':ti,ab,kw,de,dn,df,mn,tn OR 'enamels':ti,ab,kw,de,dn,df,mn,tn OR 'dental hard tissue':ti,ab,kw,de,dn,df,mn,tn	45969	
	#03	tooth demineralization'/exp OR 'demineralization tooth':ti,ab,kw,de,dn,df,mn,tn OR 'decalcification*':ti,ab,kw,de,dn,df,mn,tn OR 'demineral i*':ti,ab,kw,de,dn,df,mn,tn OR 'dental demineralization':ti,ab,kw,de,dn,df,mn,tn	90171	
	#04	dental erosion'/exp OR 'erosion tooth':ti,ab,kw,de,dn,df,mn,tn OR 'tooth erosion*':ti,ab,kw,de,dn,df,mn,tn OR 'dental erosion':ti,ab,kw,de,dn,df,mn,tn OR 'dental erosions':ti,ab,kw,de,dn,df,mn,tn OR 'erosion dental':ti,ab,kw,de,dn,df,mn,tn OR 'dental enamel erosion':ti,ab,kw,de,dn,df,mn,tn OR 'dental enamel erosions':ti,ab,kw,de,dn,df,mn,tn OR 'enamel erosion':ti,ab,kw,de,dn,df,mn,tn OR 'erosion dental enamel':ti,ab,kw,de,dn,df,mn,tn OR 'eroded teeth':ti,ab,kw,de,dn,df,mn,tn OR 'eroded tooth':ti,ab,kw,de,dn,df,mn,tn	2519	
	#05	collagen type 1'/exp OR 'type 1 collagen':ti,ab,kw,de,dn,df,mn,tn OR 'collagen type 1':ti,ab,kw,de,dn,df,mn,tn OR 'type i collagen':ti,ab,kw,de,dn,df,mn,tn OR 'collagen type i':ti,ab,kw,de,dn,df,mn,tn OR 'collagen 1':ti,ab,kw,de,dn,df,mn,tn OR 'collgen i':ti,ab,kw,de,dn,df,mn,tn OR 'collagen type 1':ti,ab,kw,de,dn,df,mn,tn	89374	
	#06	gelatinase B'/exp OR 'metalloproteinase 9 matrix':ti,ab,kw,de,dn,df,mn,tn OR '92 kda type iv collagenase':ti,ab,kw,de,dn,df,mn,tn OR '92 kda type iv collagenase':ti,ab,kw,de,dn,df,mn,tn OR 'matrix metalloproteinase 9':ti,ab,kw OR '92 kda gelatinase':ti,ab,kw,de,dn,df,mn,tn OR 'mmp 9 metalloproteinase':ti,ab,kw,de,dn,df,mn,tn OR 'metalloproteinase mmp 9':ti,ab,kw,de,dn,df,mn,tn OR 'mmp 9 metalloproteinase':ti,ab,kw,de,dn,df,mn,tn OR 'mmp9 metalloproteinase':ti,ab,kw,de,dn,df,mn,tn OR	83521	

		'metalloproteinase mmp9':ti,ab,kw,de,dn,df,mn,tn OR 'gelatinase b':ti,ab,kw,de,dn,df,mn,tn OR 'mmp9':ti,ab,kw,de,dn,df,mn,tn OR 'mmp-9':ti,ab,kw OR 'mmp-9':ti,ab,kw,de,dn,df,mn,tn		
	#07	gelatinase A'/exp OR 'metalloproteinase 2 matrix':ti,ab,kw,de,dn,df,mn,tn OR '72 kda type iv collagenase':ti,ab,kw,de,dn,df,mn,tn OR '72 kda type iv collagenase':ti,ab,kw,de,dn,df,mn,tn OR 'matrix metalloproteinase 2':ti,ab,kw OR '72 kda gelatinase':ti,ab,kw,de,dn,df,mn,tn OR 'gelatinase 72 kda':ti,ab,kw,de,dn,df,mn,tn OR 'mmp 2 metalloproteinase':ti,ab,kw,de,dn,df,mn,tn OR 'metalloproteinase mmp 2':ti,ab,kw,de,dn,df,mn,tn OR 'mmp 2 metalloproteinase':ti,ab,kw,de,dn,df,mn,tn OR 'mmp2 metalloproteinase':ti,ab,kw,de,dn,df,mn,tn OR 'metalloproteinase mmp2':ti,ab,kw,de,dn,df,mn,tn OR 'gelatinase a':ti,ab,kw,de,dn,df,mn,tn OR 'mmp-2':ti,ab,kw OR 'mmp2':ti,ab,kw OR 'type iv collagenase':ti,ab,kw,de,dn,df,mn,tn	60249	
	#08	collagenase'/exp OR 'collagenase*':ti,ab,kw OR 'collagen peptidase':ti,ab,kw,de,dn,df,mn,tn OR 'collagen degrading enzyme':ti,ab,kw,de,dn,df,mn,tn OR 'nucleolysin':ti,ab,kw,de,dn,df,mn,tn	37295	
	#09	gelatinase'/exp OR 'gelatinase':ti,ab,kw,de,dn,df,mn,tn OR 'gelatinases':ti,ab,kw,de,dn,df,mn,tn	115486	
	#10	#1 OR #2 OR #3 OR #4 OR #5 OR #6 OR #7 OR #8 OR #9	**764267**	
	#11	phlorotannin*':ti,ab,kw,de,dn,df,mn,tn OR 'phlorotannin*':ti,ab,kw	765	C
	#12	fucophlorethol*':ti,ab,kw,de,dn,df,mn,tn OR 'fucophlorethol*':ti,ab,kw	19	
	#13	eckol*':ti,ab,kw,de,dn,df,mn,tn OR 'eckol*':ti,ab,kw	170	
	#14	fucan sulfate*':ti,ab,kw,de,dn,df,mn,tn OR 'fucoidin*':ti,ab,kw,de,dn,df,mn,tn OR 'sulfated fucan*':ti,ab,kw,de,dn,df,mn,tn OR 'fucan sulfate hor 1':ti,ab,kw,de,dn,df,mn,tn OR 'mekabu fucoidan':ti,ab,kw,de,dn,df,mn,tn OR 'mefucoidan':ti,ab,kw,de,dn,df,mn,tn OR 'fucoidan*':ti,ab,kw,de,dn,df,mn,tn OR 'fucan sulfate*':ti,ab,kw OR 'fucoidin*':ti,ab,kw OR 'fucoidan*':ti,ab,kw	4654	
	#15	laminarin*':ti,ab,kw,de,dn,df,mn,tn OR 'laminaran*':ti,ab,kw,de,dn,df,mn,tn OR 'laminarin*':ti,ab,kw OR 'laminaran*':ti,ab,kw	2013	
	#16	phytosterol'/exp OR 'plant sterol*':ti,ab,kw,de,dn,df,mn,tn OR 'sterol plant':ti,ab,kw,de,dn,df,mn,tn OR 'plant steroid*':ti,ab,kw,de,dn,df,mn,tn OR 'steroid plant':ti,ab,kw,de,dn,df,mn,tn OR 'phytosteroid*':ti,ab,kw,de,dn,df,mn,tn OR 'sterols plant':ti,ab,kw,de,dn,df,mn,tn OR	13310	

		'phytosterol*':ti,ab,kw,de,dn,df,mn,tn OR 'steroids plant':ti,ab,kw,de,dn,df,mn,tn		
	#17	fucoxanthin*':ti,ab,kw,de,dn,df,mn,tn OR 'fucoxanthin*':ti,ab,kw	1961	
	#18	avenasterol*':ti,ab,kw,de,dn,df,mn,tn OR '28-isofucosterol':ti,ab,kw,de,dn,df,mn,tn OR 'fucosterol*':ti,ab,kw,de,dn,df,mn,tn OR 'avenasterol*':ti,ab,kw OR 'fucosterol*':ti,ab,kw	657	
	#19	brown alga'/exp OR 'phaeophyta*':ti,ab,kw,de,dn,df,mn,tn OR 'algae brown':ti,ab,kw,de,dn,df,mn,tn OR 'brown alga*':ti,ab,kw,de,dn,df,mn,tn OR 'fucophyceae':ti,ab,kw,de,dn,df,mn,tn OR 'phaeophyceae*':ti,ab,kw,de,dn,df,mn,tn OR 'phaeophyt*':ti,ab,kw,de,dn,df,mn,tn OR 'seaweed*':ti,ab,kw,de,dn,df,mn,tn	19653	
	#20	alga*':ti,ab,kw,de,dn,df,mn,tn	90819	
	#21	Undaria'/exp OR 'wakame*':ti,ab,kw,de,dn,df,mn,tn OR 'alginate*':ti,ab,kw,de,dn,df,mn,tn	39664	
	#22	kelp'/exp OR 'saccharina japonica':ti,ab,kw,de,dn,df,mn,tn OR 'laminaria japonica':ti,ab,kw,de,dn,df,mn,tn OR 'konbu kelp*':ti,ab,kw,de,dn,df,mn,tn OR 'dasima*':ti,ab,kw,de,dn,df,mn,tn OR 'haidai*':ti,ab,kw,de,dn,df,mn,tn OR 'kombu*':ti,ab,kw,de,dn,df,mn,tn OR 'kjellmaniella':ti,ab,kw,de,dn,df,mn,tn OR 'saccharina':ti,ab,kw,de,dn,df,mn,tn OR 'miyeok*':ti,ab,kw,de,dn,df,mn,tn OR 'kelp*':ti,ab,kw,de,dn,df,mn,tn OR 'konbu*':ti,ab,kw,de,dn,df,mn,tn	3974	
	#23	seaweed'/exp OR 'macroalgae':ti,ab,kw,de,dn,df,mn,tn	11285	
	#24	green alga'/exp OR 'green algae':ti,ab,kw,de,dn,df,mn,tn OR 'algae green':ti,ab,kw,de,dn,df,mn,tn OR 'chlorophytina':ti,ab,kw,de,dn,df,mn,tn	38836	
	#25	red alga'/exp OR 'algae red':ti,ab,kw,de,dn,df,mn,tn OR 'red algae':ti,ab,kw,de,dn,df,mn,tn	7673	
	#26	carrageenan'/exp OR 'carrageenin':ti,ab,kw,de,dn,df,mn,tn OR 'iota-carrageenan':ti,ab,kw,de,dn,df,mn,tn OR 'iota-carrageenan':ti,ab,kw,de,dn,df,mn,tn OR 'kappa-carrageenan':ti,ab,kw,de,dn,df,mn,tn OR 'kappa-carrageenan':ti,ab,kw,de,dn,df,mn,tn OR 'lambda-carrageenan':ti,ab,kw,de,dn,df,mn,tn OR 'lambda-carrageenan':ti,ab,kw,de,dn,df,mn,tn	16955	
	#27	#11 OR #12 OR #13 OR #14 OR #15 OR #16 OR #17 OR #18 OR #19 OR #20 OR #21 OR #22 OR #23 OR #24 OR #25 OR #26	**157315**	
	#28	dentin bonding*':ti,ab,kw,de,dn,df,mn,tn	7858	**Cx**
	#29	dentin bonding agent'/exp OR 'dentin bonding agent*':ti,ab,kw,de,dn,df,mn,tn	7431	

	#30	bond strength*':ti,ab,kw,de,dn,df,mn,tn	15207	
	#31	matrix metalloproteinase inhibitor'/exp OR 'mmp inhibitor*':ti,ab,kw,de,dn,df,mn,tn OR 'collagenase inhibitor*':ti,ab,kw,de,dn,df,mn,tn OR 'gelatinase inhibitor*':ti,ab,kw,de,dn,df,mn,tn OR 'stromelysin inhibitor*':ti,ab,kw,de,dn,df,mn,tn OR 'matrix metalloproteinase inhibitor*':ti,ab,kw,de,dn,df,mn,tn OR 'mmp inhibit*':ti,ab,kw,de,dn,df,mn,tn	44125	
	#32	dental procedure'/exp OR 'tooth remineralization*':ti,ab,kw,de,dn,df,mn,tn OR 'remineraliz*':ti,ab,kw,de,dn,df,mn,tn OR 'remineralis*':ti,ab,kw,de,dn,df,mn,tn	290398	
	#33	collagen crosslink*':ti,ab,kw,de,dn,df,mn,tn OR 'crosslink*':ti,ab,kw,de,dn,df,mn,tn	159439	
	#34	#28 OR #29 OR #30 OR #31 OR #32 OR #33	**498837**	
	#35	#10 AND #27 AND #34	**790**	
	#36	bone'/exp OR ('bone':ti,ab,kw,de,dn,df,mn,tn AND 'bone':ti,ab,kw,de,dn,df,mn,tn) OR ('bones':ti,ab,kw,de,dn,df,mn,tn AND 'bone':ti,ab,kw,de,dn,df,mn,tn) OR ('bones':ti,ab,kw,de,dn,df,mn,tn AND 'bone tissue':ti,ab,kw,de,dn,df,mn,tn) OR 'bones':ti,ab,kw,de,dn,df,mn,tn OR 'bone':ti,ab,kw,de,dn,df,mn,tn OR 'bone tissue':ti,ab,kw,de,dn,df,mn,tn OR 'bone tissues':ti,ab,kw,de,dn,df,mn,tn OR 'tissue bone':ti,ab,kw,de,dn,df,mn,tn OR 'tissues bone':ti,ab,kw,de,dn,df,mn,tn OR 'bony apophyses':ti,ab,kw,de,dn,df,mn,tn OR (('apophyseal' OR 'apophyses' OR 'apophysitis') AND 'bony':ti,ab,kw,de,dn,df,mn,tn) OR ('bony' AND 'apophysis':ti,ab,kw,de,dn,df,mn,tn) OR ('apophysis' AND 'bony':ti,ab,kw,de,dn,df,mn,tn) OR 'condyle':ti,ab,kw,de,dn,df,mn,tn OR 'condyles':ti,ab,kw,de,dn,df,mn,tn	2397359	**Lim**
	#37	hydrogel'/exp OR 'hydrogel':ti,ab,kw,de,dn,df,mn,tn OR 'in situ hydrogels':ti,ab,kw,de,dn,df,mn,tn OR 'in situ hydrogel':ti,ab,kw,de,dn,df,mn,tn OR 'hydrogel in situ':ti,ab,kw,de,dn,df,mn,tn OR 'patterned hydrogels':ti,ab,kw,de,dn,df,mn,tn OR 'patterned hydrogel':ti,ab,kw,de,dn,df,mn,tn OR 'hydrogel patterned':ti,ab,kw,de,dn,df,mn,tn	99724	
	#38	cartilage'/exp OR 'cartilages':ti,ab,kw,de,dn,df,mn,tn	192193	
	#39	#35 NOT #36 NOT #37 NOT #38	**449**	

Table S3. Scopus Search
Database: Scopus All to November 1, 2025
Date searched: November 1, 2025

	#	Query	Results	P/C/Cx
	#01	INDEXTERMS(dentin) OR TITLE-ABS(dentin) OR TITLE-ABS-KEY(dentin) OR TITLE-ABS-KEY(Dentine) OR TITLE-ABS-KEY(Dentines) OR TITLE-ABS-KEY(dentinal) OR TITLE-ABS-KEY(dentinum) OR TITLE-ABS(tooth) OR TITLE-ABS(teeth) OR TITLE-ABS(dental)	617826	
	#02	INDEXTERMS("dental enamel") OR TITLE-ABS-KEY(Enamel) OR TITLE-ABS-KEY(Enamels) OR TITLE-ABS-KEY("dental hard tissue")	68589	
	#03	INDEXTERMS("tooth demineralization") OR TITLE-ABS-KEY("demineralization tooth") OR TITLE-ABS-KEY(decalcification*) OR TITLE-ABS-KEY(deminerali*) OR TITLE-ABS-KEY("dental demineralization")	30172	
	#04	INDEXTERMS("tooth erosion") OR TITLE-ABS-KEY("erosion tooth") OR TITLE-ABS-KEY("tooth erosion*") OR TITLE-ABS-KEY("dental erosion") OR TITLE-ABS-KEY("dental erosions") OR TITLE-ABS-KEY("erosion dental") OR TITLE-ABS-KEY("dental enamel erosion") OR TITLE-ABS-KEY("dental enamel erosions") OR TITLE-ABS-KEY("enamel erosion") OR TITLE-ABS-KEY("erosion dental enamel") OR TITLE-ABS-KEY("eroded teeth") OR TITLE-ABS-KEY("eroded tooth")	4517	
**Scopus**	#05	INDEXTERMS("collagen type i") OR TITLE-ABS-KEY("type 1 collagen") OR TITLE-ABS-KEY("collagen type 1") OR TITLE-ABS-KEY("type i collagen") OR TITLE-ABS-KEY("collagen type i") OR TITLE-ABS-KEY("collagen 1") OR TITLE-ABS-KEY("collgen i") OR TITLE-ABS-KEY("collagen type 1")	78694	**P**
	#06	INDEXTERMS("matrix metalloproteinase 9") OR TITLE-ABS-KEY("metalloproteinase 9 matrix") OR TITLE-ABS-KEY("92 kda type iv collagenase") OR TITLE-ABS-KEY("92 kda type iv collagenase") OR TITLE-ABS("matrix metalloproteinase 9") OR TITLE-ABS-KEY("92 kda gelatinase") OR TITLE-ABS-KEY("mmp 9 metalloproteinase") OR TITLE-ABS-KEY("metalloproteinase mmp 9") OR TITLE-ABS-KEY("mmp 9 metalloproteinase") OR TITLE-ABS-KEY("mmp9 metalloproteinase") OR TITLE-ABS-KEY("metalloproteinase mmp9") OR TITLE-ABS-KEY("gelatinase b") OR TITLE-ABS-KEY(MMP9) OR TITLE-ABS(MMP-9) OR TITLE-ABS-KEY(MMP-9)	73359	
	#07	INDEXTERMS("matrix metalloproteinase 2") OR TITLE-ABS-KEY("metalloproteinase 2 matrix") OR TITLE-ABS-KEY("72 kda type iv collagenase") OR TITLE-ABS-KEY("72 kda type iv collagenase") OR TITLE-ABS("matrix metalloproteinase 2") OR TITLE-ABS-KEY("72 kda gelatinase") OR TITLE-ABS-KEY("gelatinase 72 kda") OR	54118	

		TITLE-ABS-KEY("mmp 2 metalloproteinase") OR TITLE-ABS-KEY("metalloproteinase mmp 2") OR TITLE-ABS-KEY("mmp 2 metalloproteinase") OR TITLE-ABS-KEY("mmp2 metalloproteinase") OR TITLE-ABS-KEY("metalloproteinase mmp2") OR TITLE-ABS-KEY("gelatinase a") OR TITLE-ABS(MMP-2) OR TITLE-ABS(MMP2) OR TITLE-ABS-KEY("type iv collagenase")		
	#08	INDEXTERMS(collagenases) OR TITLE-ABS(collagenase*) OR TITLE-ABS-KEY("collagen peptidase") OR TITLE-ABS-KEY("collagen degrading enzyme") OR TITLE-ABS-KEY(nucleolysin)	29957	
	#09	INDEXTERMS(gelatinases) OR TITLE-ABS-KEY(Gelatinase) OR TITLE-ABS-KEY(gelatinases)	98637	
	#10	#1 OR #2 OR #3 OR #4 OR #5 OR #6 OR #7 OR #8 OR #9	865640	
	#11	TITLE-ABS-KEY(phlorotannin*) OR TITLE-ABS(phlorotannin*)	1458	**C**
	#12	TITLE-ABS-KEY(fucophlorethol*) OR TITLE-ABS(fucophlorethol*)	44	
	#13	TITLE-ABS-KEY(eckol*) OR TITLE-ABS(eckol*)	234	
	#14	TITLE-ABS-KEY("fucan sulfate*") OR TITLE-ABS-KEY(fucoidin*) OR TITLE-ABS-KEY("sulfated fucan*") OR TITLE-ABS-KEY("fucan sulfate hor 1") OR TITLE-ABS-KEY("mekabu fucoidan") OR TITLE-ABS-KEY(meFucoidan) OR TITLE-ABS-KEY(fucoidan*) OR TITLE-ABS("fucan sulfate*") OR TITLE-ABS(fucoidin*) OR TITLE-ABS(fucoidan*)	5939	
	#15	TITLE-ABS-KEY(laminarin*) OR TITLE-ABS-KEY(laminaran*) OR TITLE-ABS(laminarin*) OR TITLE-ABS(laminaran*)	2979	
	#16	INDEXTERMS(phytosterols) OR TITLE-ABS-KEY("plant sterol*") OR TITLE-ABS-KEY("sterol plant") OR TITLE-ABS-KEY("plant steroid*") OR TITLE-ABS-KEY("steroid plant") OR TITLE-ABS-KEY(phytosteroid*) OR TITLE-ABS-KEY("sterols plant") OR TITLE-ABS-KEY(phytosterol*) OR TITLE-ABS-KEY("steroids plant")	17194	
	#17	TITLE-ABS-KEY(fucoxanthin*) OR TITLE-ABS(fucoxanthin*)	3496	
	#18	TITLE-ABS-KEY(avenasterol*) OR TITLE-ABS-KEY(28-isofucosterol) OR TITLE-ABS-KEY(fucosterol*) OR TITLE-ABS(avenasterol*) OR TITLE-ABS(fucosterol*)	1204	
	#19	INDEXTERMS(phaeophyceae) OR TITLE-ABS-KEY(phaeophyta*) OR TITLE-ABS-KEY("algae brown") OR TITLE-ABS-KEY("brown alga*") OR TITLE-ABS-KEY(fucophyceae) OR TITLE-ABS-KEY(phaeophyceae*) OR TITLE-ABS-KEY(phaeophyt*) OR TITLE-ABS-KEY(seaweed*)	45626	
	#20	TITLE-ABS-KEY (alga*)	303783	

	#21	INDEXTERMS(undaria) OR TITLE-ABS-KEY(wakame*) OR TITLE-ABS-KEY(alginate*)	69750	
	#22	INDEXTERMS(kelp) OR TITLE-ABS-KEY("saccharina japonica") OR TITLE-ABS-KEY("laminaria japonica") OR TITLE-ABS-KEY("konbu kelp*") OR TITLE-ABS-KEY(dasima*) OR TITLE-ABS-KEY(haidai*) OR TITLE-ABS-KEY(kombu*) OR TITLE-ABS-KEY(Kjellmaniella) OR TITLE-ABS-KEY(Saccharina) OR TITLE-ABS-KEY(miyeok*) OR TITLE-ABS-KEY(kelp*) OR TITLE-ABS-KEY(konbu*)	12490	
	#23	INDEXTERMS(seaweed) OR TITLE-ABS-KEY(Macroalgae)	29759	
	#24	INDEXTERMS(chlorophyta) OR TITLE-ABS-KEY("green algae") OR TITLE-ABS-KEY("algae green") OR TITLE-ABS-KEY(Chlorophytina)	42761	
	#25	INDEXTERMS(rhodophyta) OR TITLE-ABS-KEY("algae red") OR TITLE-ABS-KEY("red algae")	16570	
	#26	INDEXTERMS(Carrageenan) OR TITLE-ABS-KEY(Carrageenin) OR TITLE-ABS-KEY(iota-Carrageenan) OR TITLE-ABS-KEY(iota-Carrageenan) OR TITLE-ABS-KEY(kappa-Carrageenan) OR TITLE-ABS-KEY(kappa-Carrageenan) OR TITLE-ABS-KEY(lambda-Carrageenan) OR TITLE-ABS-KEY(lambda-Carrageenan)	22601	
	#27	#11 OR #12 OR #13 OR #14 OR #15 OR #16 OR #17 OR #18 OR #19 OR #20 OR #21 OR #22 OR #23 OR #24 OR #25 OR #26	446058	
	#28	TITLE-ABS-KEY("dentin bonding*")	8349	**Cx**
	#29	INDEXTERMS("dentin bonding agents") OR TITLE-ABS-KEY("dentin bonding agent*")	7719	
	#30	TITLE-ABS-KEY("bond strength*")	70529	
	#31	INDEXTERMS("matrix metalloproteinase inhibitors") OR TITLE-ABS-KEY("mmp inhibitor*") OR TITLE-ABS-KEY("collagenase inhibitor*") OR TITLE-ABS-KEY("gelatinase inhibitor*") OR TITLE-ABS-KEY("stromelysin inhibitor*") OR TITLE-ABS-KEY("matrix metalloproteinase inhibitor*") OR TITLE-ABS-KEY("mmp inhibit*")	9548	
	#32	INDEXTERMS("Tooth Remineralization") OR TITLE-ABS-KEY("tooth remineralization*") OR TITLE-ABS-KEY(remineraliz*) OR TITLE-ABS-KEY(remineralis*)	11042	
	#33	TITLE-ABS-KEY("collagen crosslink*") OR TITLE-ABS-KEY(crosslink*)	205984	
	#34	#28 OR #29 OR #30 OR #31 OR #32 OR #33	299175	
	#35	#10 AND #27 AND #34	216	
	#36	INDEXTERMS("bone and bones") OR (TITLE-ABS-KEY(Bone) AND TITLE-ABS-KEY(Bone)) OR (TITLE-ABS-KEY(Bones) AND TITLE-ABS-KEY(Bone)) OR (TITLE-ABS-KEY(Bones) AND TITLE-ABS-KEY("bone tissue")) OR TITLE-ABS-KEY(Bones) OR TITLE-ABS-	1837863	**Lim**

		KEY(Bone) OR TITLE-ABS-KEY("bone tissue") OR TITLE-ABS-KEY("bone tissues") OR TITLE-ABS-KEY("tissue bone") OR TITLE-ABS-KEY("tissues bone") OR TITLE-ABS-KEY("bony apophyses") OR ((ALL(apophyseal) OR ALL(Apophyses) OR ALL(apophysitis)) AND TITLE-ABS-KEY(Bony)) OR (ALL(Bony) AND TITLE-ABS-KEY(Apophysis)) OR (ALL(Apophysis) AND TITLE-ABS-KEY(Bony)) OR TITLE-ABS-KEY(Condyle) OR TITLE-ABS-KEY(Condyles)		
	#37	INDEXTERMS(Hydrogels) OR TITLE-ABS-KEY(Hydrogel) OR TITLE-ABS-KEY("in situ hydrogels") OR TITLE-ABS-KEY("in situ hydrogel") OR TITLE-ABS-KEY("hydrogel in situ") OR TITLE-ABS-KEY("patterned hydrogels") OR TITLE-ABS-KEY("patterned hydrogel") OR TITLE-ABS-KEY("hydrogel patterned")	169079	
	#38	INDEXTERMS(cartilage) OR TITLE-ABS-KEY(Cartilages)	188762	
	#39	#35 NOT #36 NOT #37 NOT #38	**107**	

Table S4. Web of Science Search
Database: Web of Science All to November 1, 2025
Date searched: November 1, 2025

	#	Query	Results	P/C/Cx
**WOS**	#01	TS=dentin* OR TS=tooth OR TS=teeth OR TS=dental	449850	**P**
	#02	TS=("dental enamel" OR Enamel OR Enamels OR "dental hard tissue")	50591	
	#03	TS=("tooth demineralization" OR "demineralization tooth" OR decalcification* OR deminerali* OR "dental demineralization")	21690	
	#04	TS=("tooth erosion" OR "erosion tooth" OR "tooth erosion*" OR "dental erosion" OR "dental erosions" OR "erosion dental" OR "dental enamel erosion" OR "dental enamel erosions" OR "enamel erosion" OR "erosion dental enamel" OR "eroded teeth" OR "eroded tooth")	2551	
	#05	TS=("collagen type i" OR "type 1 collagen" OR "collagen type 1" OR "type i collagen" OR "collagen type i" OR "collagen 1" OR "collgen i" OR "collagen type 1")	35812	
	#06	TS=("matrix metalloproteinase 9" OR "metalloproteinase 9 matrix" OR "92 kda type iv collagenase" OR "92 kda type iv collagenase" OR "92 kda gelatinase" OR "mmp 9 metalloproteinase" OR "metalloproteinase mmp 9" OR "mmp 9 metalloproteinase" OR "mmp9 metalloproteinase" OR "metalloproteinase mmp9" OR "gelatinase b" OR MMP9 OR MMP-9) OR TS="matrix metalloproteinase 9" OR TS=MMP-9	47918	
	#07	TS=("matrix metalloproteinase 2" OR "metalloproteinase 2 matrix" OR "72 kda type iv collagenase" OR "72 kda type iv collagenase" OR "72 kda gelatinase" OR "gelatinase 72 kda" OR "mmp 2 metalloproteinase" OR "metalloproteinase mmp 2" OR "mmp 2 metalloproteinase" OR "mmp2 metalloproteinase" OR "metalloproteinase mmp2" OR "gelatinase a" OR "type iv collagenase" OR "type iv collagenase") OR TS=MMP-2 OR TS=MMP2 OR TS="matrix metalloproteinase 2"	35800	
	#08	TS=(collagenases OR "collagen peptidase" OR "collagen degrading enzyme" OR nucleolysin) OR TS=collagenase*	32535	
	#09	TS=(gelatinases OR Gelatinase)	18764	
	#10	#7 OR #6 OR #5 OR #4 OR #3 OR #2 OR #1	595557	
	#11	TS=phlorotannin*	1707	**C**
	#12	TS=fucophlorethol*	50	
	#13	TS=eckol*	189	
	#14	TS=("fucan sulfate*" OR fucoidin* OR "sulfated fucan*" OR "fucan sulfate hor 1" OR "mekabu fucoidan" OR meFucoidan OR fucoidan*)	5776	
	#15	TS=laminarin* OR TS=laminaran*	2560	

	#16	TS=(phytosterols OR "plant sterol*" OR "sterol plant" OR "plant steroid*" OR "steroid plant" OR phytosteroid* OR "sterols plant" OR phytosterol* OR "steroids plant")	11480	
	#17	TS=fucoxanthin*	3157	
	#18	TS=(avenasterol* OR 28-isofucosterol OR fucosterol*)	841	
	#19	TS=(phaeophyceae OR phaeophyta* OR "algae brown" OR "brown alga*" OR fucophyceae OR phaeophyceae* OR phaeophyt* OR seaweed*)	39023	
	#20	TS=alga*	246690	
	#21	TS=(undaria OR wakame* OR alginate*)	68563	
	#22	TS=(kelp OR "saccharina japonica" OR "laminaria japonica" OR "konbu kelp*" OR dasima* OR haidai* OR kombu* OR Kjellmaniella OR Saccharina OR miyeok* OR kelp* OR konbu*)	13094	
	#23	TS=(seaweed OR Macroalgae)	41266	
	#24	TS=(chlorophyta OR "green algae" OR "algae green" OR Chlorophytina)	27856	
	#25	TS=(rhodophyta OR "algae red" OR "red algae")	14808	
	#26	TS=(Carrageenan OR Carrageenin OR iota-Carrageenan ORiota-Carrageenan OR kappa-Carrageenan OR kappa-Carrageenan OR lambda-Carrageenan OR lambda-Carrageenan)	23707	
	#27	#20 OR #19 OR #18 OR #17 OR #16 OR #15 OR #14 OR #13 OR #12 OR #11 OR #10 OR #9	353145	
	#28	TS="dentin bonding*"	2185	
	#29	TS=("dentin bonding agents" OR "dentin bonding agent*")	898	
	#30	TS="bond strength*"	42728	
	#31	TS=("matrix metalloproteinase inhibitors" OR "mmp inhibitor*" OR "collagenase inhibitor*" OR "gelatinase inhibitor*" OR "stromelysin inhibitor*" OR "matrix metalloproteinase inhibitor*" OR "mmp inhibit*")	6308	**Cx**
	#32	TS=("Tooth Remineralization" OR "tooth remineralization*" OR remineraliz* OR remineralis*)	10072	
	#33	TS=("collagen crosslink*" OR crosslink*)	135384	
	#34	#27 OR #26 OR #25 OR #24 OR #23 OR #22	194511	
	#35	#28 AND #21 AND #8	128	
	#36	TS=("bone and bones" OR (TS=Bone AND TS=Bone) OR (TS=Bones AND TS=Bone) OR (TS=Bones AND TS="bone tissue") OR Bones OR Bone OR "bone tissue" OR "bone tissues" OR "tissue bone" OR "tissues bone" OR "bony apophyses" OR ((TS=(apophyseal OR Apophyses OR apophysitis) AND Bony) OR (TS=Bony AND Apophysis) OR (TS=Apophysis AND Bony) OR Condyle OR Condyles	1202383	**Lim**
	#37	TS=(Hydrogels OR Hydrogel OR"in situ hydrogels" OR "in situ hydrogel" OR "hydrogel in situ" OR "patterned hydrogels" OR "patterned hydrogel" OR "hydrogel patterned")	184544	

	#38	TS=(cartilage OR Cartilages)	143226	
	#39	#35 NOT #36 NOT #37 NOT #38	49	

Table S5. **Population inclusion and exclusion criteria with examples**

Inclusion	Population	Examples
Include	Human or animal dental hard-tissues (enamel, dentin) or collagen-based dentin model	- Collagen film or dentin matrix model (ex vivo or in vitro)<br>- Extracted human/bovine enamel or dentin
Exclude	Non-dental hard-tissues or non-tooth substrates	- Bone regeneration, cartilage, skin, liver fibrosis models
Exclude	no enamel or dentin/collagen component	- Resin composite specimen only, bioceramic bulk discs
Exclude	Endodontic or pulp-only studies	- Root canal irrigants, apex locators, pulp regeneration using alginate hydrogel
Exclude	non-tooth cell lines only	-HT1080 fibrosarcoma cell MMP assay without tooth substrate

Table S6. **Concept inclusion and exclusion criteria with examples.**

Inclusion	Population	Examples
Include	Algae-derived bioactive compounds (from brown, red, or green algae) and the effects on the structural stability of dental hard tissues	- Fucoxanthin, fucosterol, fucoidan, laminarin, phlorotannins, eckol, alginate, sulfated fucans<br>- major mechanisms include but not limited to : remineralization inhibition, collagen stabilization, MMP inhibition, increase in collagen cross-links, increase in bond stability
Exclude	Compounds not derived from algae	- Compounds derived from organisms other than brown, red, green algae (e.g: chitosan from crustaceans or fungi, synthetic crosslinkers)
Exclude	Source of algae not identified or uncertain	-“Marine extract” only, species unspecified
Exclude	Algae used as carrier or binder only	- Alginate impression materials, alginate drug films, bone scaffolds
Exclude	No relevant mechanistic outcome	- No assessment of demineralization, remineralization, MMP, crosslink, microhardness etc.
Exclude	Delivery-only nanoparticle studies without dental hard-tissue outcomes	- Nanoparticle system evaluated only for drug release, cytotoxicity, or systemic delivery — no enamel/dentin data

Table S7. **Context inclusion and exclusion criteria with examples.**

Inclusion	Population	Examples
Include	Experimental or pre-clininical research within the field of dentistry, dental materials, and oral hard-tissue biomodification	- Conservative dentistry, adhesive dentistry, enamel erosion, remineralization, MMP activation/inhibition control<br>- include all in vitro, ex vivo, in vivo
Exclude	Non-dental field or non-oral application	- Marine-derived compound applied to dermatology or pharmaceutical models only
Exclude	Clinical procedural or device focus	- Orthodontic bracket bonding, laser curing, whitening, prosthodontic fabrication
Exclude	Publication type not original research	- Review, conference abstract, book section, editorial, case report
Exclude	Language or access limitation	- Non-English or full text unavailable
Exclude	Duplicate dataset	- Reporting same redundant experimental results

Table S8. **Main themes to be studied with description.**

Inclusion	Type	Theme	Examples
Include	Primary	Dimineralization inhibtion/ Remineralization	- Studies evaluating mineral recovery, Ca/P uptake, surface microhardness, and enamel subsurface lesion mineralization underacid or pH-cycling conditions
Include	Primary	Collagen stabilization/ crosslinking enhancement	- Investigations of dentin collagen matrix stabilization via cross-link formation, FTIR spectral shifts or reduced enzymatic degradation
Include	Primary	Matrix Metalloproteinase (MMP) inhibition	- Studies targeting MMP-2 and MMP-9 activity reduction in dentin or hybrid layer, using in-situ zymography or gelatinase assays
Include	Primary	Erosion and surface protection	- Evaluation of seaweed-derived polysaccharides forming protective films or increasing acid resistance of enamel/dentin
Include	Secondary	Bond strength and hybrid layer durability	- Assessment of long-term bond strength, nanoleakage, and hydrolytic degradation prevention at the resin-dentin interface
